# Principles of Surgery

**Published:** 1891-06

**Authors:** 


### Reviews of Books.

*Principles of Surgery*. By N. SENN, M.D. Pp. 611. Philadelphia: F. A. DAVIS. 1890.

For several reasons the book before us is worthy of more than the passing notice we can give to it. It is the work of one of the ablest and best known among the younger generation of American surgeons. It marks a new departure in the presentment of a large body of medical knowledge. And finally, in numerous instances, doctrines are enunciated which are not in keeping with those ordinarily taught.

To take the last point first. It is not our intention to offer any detailed criticism on views which many of us would consider erroneous or improved. Right or wrong, they are always philosophic and plausible; and even where they seem to be most faulty, they are full of suggestive, almost startling, novelty, and a study of them can only do good. Senn is a man who thinks for himself: this attribute alone should make him a welcome, if novel, addition to the host of writing surgeons.

The work is distinctly a new departure in surgical literature. It deals with the "fundamental principles of the art and science of surgery," according to the lights of modern research. The healing of wounds and the behaviour of diseased tissues are studied from every possible point of view, both as regards special tissues and special processes. The influence of bacteria on wounds receives, almost for the first time in a work on general surgery, the full measure of attention which its supreme importance demands. Among diseases of wound infection are properly ranged, not only Septicæmia and Pyæmia, and Erysipelas; but also Tetanus, Hydrophobia, Actinomycosis, Anthrax, and Glanders. This is as it should be. Amongst the same class Tuberculosis holds a preponderating position.

The work is evidently that of a learned and original surgeon, and, if only as an example to others in the manner of utilising and classifying modern knowledge for teaching purposes, must have a marked influence for good. But, possibly because we expected too much, we can clearly see how easily it might have been better. The language is often heavy, and sometimes neither idiomatic nor pure; the meaning is not always clear; and the classification is occasionally open to objection.

The author, in the preface, asks our indulgence for possible defects in proof-reading, on account of his absence from home. He also tells us that "many sleepless nights were required in its preparation." In future essays, our author should remember that the public make allowance for no faults; and that the best work is done not during sleepless nights, but during waking days. Still, if he has not done himself justice, he has done us good service.

